# Smart Packaging 4.0: A Bibliometric Analysis of Sensor Integration, Food Safety, and Sustainability in Food Packaging Systems

**DOI:** 10.1155/tswj/3181510

**Published:** 2026-02-27

**Authors:** Mochammad Jusuf Djafar, Huda M. Elmatsani, S. Joni Munarso, Jonni Firdaus, Sudarwaji Edi Yuwono Trihadi, Wahyu Purwanta, Budi Setiadi Sadikin, Sahlan Sahlan, Nasruddin Nasruddin, Lanjar Lanjar

**Affiliations:** ^1^ Research Center for Horticulture, National Research and Innovation Agency, Jakarta, Indonesia, brin.go.id; ^2^ Research Center for Artificial Intelligence and Cyber Security, National Research and Innovation Agency, Jakarta, Indonesia, brin.go.id; ^3^ Research Center for Behavioral and Circular Economics, National Research and Innovation Agency, Jakarta, Indonesia, brin.go.id; ^4^ Research Center for Environmental and Clean Technology, National Research and Innovation Agency, Jakarta, Indonesia, brin.go.id; ^5^ Directorate for Scientific Collection Management, National Research and Innovation Agency, Jakarta, Indonesia, brin.go.id; ^6^ Research Center for Sustainable Industrial and Manufacturing Systems, National Research and Innovation Agency, Jakarta, Indonesia, brin.go.id; ^7^ Research Center for Equipment Manufacturing, National Research and Innovation Agency, Jakarta, Indonesia, brin.go.id; ^8^ Research Center for Process Technology, National Research and Innovation Agency, Jakarta, Indonesia, brin.go.id

**Keywords:** active packaging, bibliometric analysis, food packaging, intelligence packaging, internet of things, sensor integration, smart packaging, supply chain

## Abstract

The integration of advanced sensors and digital technologies into food packaging systems has catalyzed the emergence of Smart Packaging 4.0, transitioning the package from a passive barrier into an intelligent cyber‐physical interface for real‐time monitoring and traceability. This study employs a systematic bibliometric analysis to map the intellectual structure of the field using bibliographic data retrieved from Scopus and Web of Science, analyzing a refined dataset of 253 unique documents published between 2015 and 2024 that exhibit a robust annual publication growth rate of 30.31%. Co‐occurrence and network clustering analyses reveal three dominant research themes: (i) physicochemical sensing (pH sensors and colorimetry) for real‐time spoilage detection; (ii) active and bioactive materials (anthocyanins and chitosan) for functional food protection; and (iii) digital supply chain integration (RFID and IoT) for enhanced traceability and logistics. The technological evolution is characterized by a strategic shift from the foundations of modified atmosphere packaging in Phase I (2017–2019) and biodegradable sensor‐embedded materials in Phase II (2020–2022) to the current peak of innovation in Phase III (2023–2024), which defines Smart Packaging 4.0 through the convergence of AI, blockchain, and predictive analytics for secure and autonomous food management. Despite these advancements, critical barriers to large‐scale commercialization persist, notably nanomaterial safety concerns regarding the migration of ZnO and TiO_2_ into food, prohibitive sensor costs, and regulatory fragmentation between the European Union′s positive‐list approach and the United States′ exposure‐based model. This study provides a strategic decision‐support framework that aligns technological innovation with global sustainability mandates, offering actionable insights to guide the development of next‐generation intelligent, eco‐efficient food packaging ecosystems.

## 1. Introduction

The increasing global population, projected to reach 9.8 billion by 2050, necessitates a growing demand for a safe and stable food supply [1]. This demographic and resource pressure underscores the need for highly efficient food preservation and distribution systems, as traditional methods of preservation have become insufficient for a complex, globalized food network [2, 3].

Food packaging has served as the fundamental method for protecting food products. Its traditional function is to act as a passive barrier to protect food from physical damage, biological contamination, and deterioration [4, 5]. Packaging is crucial for ensuring food safety, preserving nutritional quality, and supporting long‐distance transportation. It contributes significantly to food security by enabling efficient global distribution and mitigating food loss, particularly reducing postharvest losses, which currently account for nearly 30% of global food waste [6]. Conventional packaging systems, relying on static barrier properties, lack the capability to provide dynamic, real‐time status updates, which are essential for managing the quality and safety of highly perishable and high‐value categories, such as meat, seafood, dairy, fruits, and ready‐to‐eat meals [7, 8].

To address these limitations, the food packaging industry is undergoing a significant transformation, driven by the comprehensive Industry 4.0 framework [9]. This technological evolution leads to the concept of Smart Packaging 4.0, where packaging systems are embedded with sensors, actuators, and communication modules that enable real‐time monitoring, traceability, and interactive functionalities [4, 10]. As a central, intelligent component of the food supply chain, smart packaging connects stakeholders from producers to consumers, facilitating precise tracking and automation of product management [11]. By providing continuous access to real‐time data, this technology helps identify potential issues early and ensures products reach consumers in optimal condition [7].

Although Smart Packaging 4.0 offers numerous advantages, existing research primarily focuses on individual technological components, such as sensors and digital technologies, without adequately addressing how these elements can be holistically integrated into a cohesive system [2, 12]. This lack of integration, coupled with challenges related to system interoperability and the complexity of managing large volumes of data, presents significant barriers to scaling the technology across industries. Issues such as data integration, real‐time traceability via Internet of Things (IoT), and the adoption of cloud or blockchain technologies remain underexplored [13, 14].

To address these gaps, this study conducts a comprehensive bibliometric analysis of the smart packaging domain, utilizing data sourced from Scopus and Web of Science. The primary objective is to map the intellectual structure of the field, identifying key technological trends, research clusters, and areas of convergence within smart packaging. Specifically, the study explores critical themes such as the integration of IoT‐enabled logistics, the development of biopolymer‐based sensors, the application of freshness indicators (FIs), and the role of consumer‐facing technologies. By synthesizing these findings, this research is aimed at providing a strategic framework for guiding future innovations in intelligent, sustainable, and digitally integrated food packaging systems, aligning closely with the principles of Industry 4.0. The derived insights are expected to guide researchers, industry stakeholders, and policymakers in advancing innovations that enhance food safety, quality, and traceability, while significantly contributing to global efforts in reducing food waste and improving sustainability within the food supply chain.

By uncovering these pivotal research areas, the study is aimed at providing a strategic overview of the technological integration within smart packaging, offering insights into how these advancements can contribute to the development of intelligent, sustainable, and digitally integrated food packaging systems. This aligns with the goals of Industry 4.0, which emphasizes automation, real‐time data exchange, and sustainability across production and distribution systems. The findings of this study are expected to guide researchers, industry stakeholders, and policymakers in fostering innovations that not only improve food safety, quality, and traceability but also contribute to reducing food waste and enhancing sustainability within the food supply chain. By linking technological components, materials, and logistics themes in a single intellectual map, this study clarifies how fragmented research streams can be integrated into a more coherent Smart Packaging 4.0 framework.

Additionally, this study seeks to address key challenges hindering the large‐scale adoption of smart packaging technologies, particularly issues related to system integration, interoperability, and data management. By examining these barriers, the research is aimed at providing actionable recommendations for overcoming technological fragmentation and optimizing the performance of smart packaging systems. These insights are essential for improving the overall efficiency, transparency, and sustainability of the food supply chain, supporting the transition to more integrated and eco‐friendly food packaging solutions. Through this approach, the study is not only aimed at advancing academic understanding of smart packaging but also seeks to offer practical frameworks for the broader implementation of these technologies, accelerating the adoption of more efficient, transparent, and sustainable smart packaging solutions across global food systems.

## 2. Materials and Methods

This study employs a systematic bibliometric approach to comprehensively map the intellectual and thematic landscape of Smart Packaging 4.0, emphasizing the integration of sensors and digital technologies in intelligent food packaging systems. The methodology is structured into three distinct yet interconnected phases.

### 2.1. Data Collection

**Figure 1:**
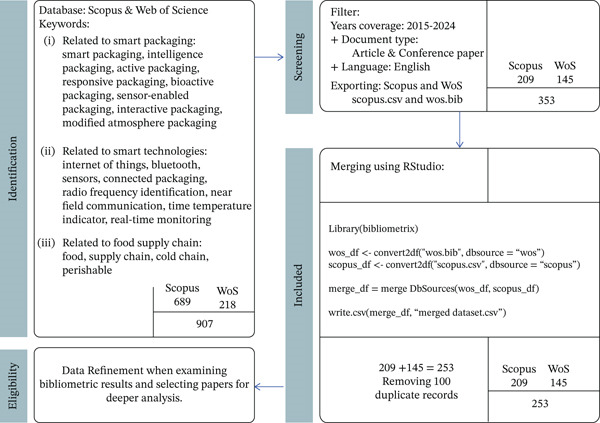
Data collection involved systematically retrieving bibliographic records from Scopus and Web of Science (WoS), followed by merging the datasets and removing duplicates using the Bibliometrix package in RStudio to ensure data consistency and reproducibility.

The data collection methodology, depicted in Figure 1, follows a structured, four‐stage bibliometric process adapted from the PRISMA framework. This process moves from broad identification to a refined final dataset using RStudio.

The first stage involves the comprehensive identification of relevant literature from two primary academic databases: Scopus and Web of Science (WoS) [15, 16]. A robust search strategy was developed to capture the interdisciplinary scope of smart and intelligent packaging technologies, covering fields such as food science, materials engineering, and digital technologies. The search query incorporated a wide range of keywords representing various technological paradigms, including “smart packaging,” “active packaging,” “intelligent packaging,” “bioactive packaging,” “responsive packaging,” and “sensor‐enabled packaging.” These keywords are recognized as core components of Smart Packaging 4.0 and reflect the ongoing digital transformation of food systems [17–21]. To ensure a comprehensive inclusion of perspectives from materials engineering and food science, the search also integrated application‐specific terms such as “food,” “supply chain,” “cold chain,” and “perishables,” thus capturing the practical context and technological needs within the food packaging sector [9, 22, 23]. Furthermore, to account for the increasing role of digital technologies, terms like “IoT,” “RFID,” “NFC,” “Bluetooth,” “real‐time monitoring,” and “connected packaging” were included, addressing the growing integration of connectivity and digital technologies in packaging innovation [6, 14, 24]. This initial broad identification phase resulted in a total of 907 documents—689 from Scopus and 218 from Web of Science.

In the second stage, a screening process was applied to refine the dataset and ensure its relevance and currency. The results were filtered to include only documents published between 2015 and 2024, focusing exclusively on articles and conference papers written in English. This filtering significantly reduced the volume of records to 353 documents—209 from Scopus and 145 from Web of Science. The refined datasets were then exported in their respective standard formats (.csv for Scopus and .bib for WoS) to facilitate computational processing.

The final phase of data preparation was conducted in the RStudio environment using the Bibliometrix R package. The raw data files were converted into dataframes and consolidated into a single dataset through the mergeDbSources function. This automated process enabled the identification and removal of approximately 100 duplicate records from both databases, resulting in a clean, unique dataset of 253 documents. Given the impracticality of manually reading the full text of such a large body of literature during the initial selection, data refinement was performed to identify papers for in‐depth analysis based on bibliometric indicators—such as citation counts, topic trends, and keyword co‐occurrences. This approach allowed for the identification of the most relevant and impactful documents, forming the basis for subsequent in‐depth qualitative review.

### 2.2. Bibliometric Analysis

The second phase of this study involves a comprehensive bibliometric analysis using two complementary tools. VOSviewer was employed to construct and visualize bibliometric networks, enabling advanced analyses such as keyword co‐occurrence, cocitation, and clustering [25]. These visualizations reveal the intellectual structure of the field by identifying key concepts, influential publications, and prominent research communities. In parallel, Bibliometrix, an R‐based software package, was used to perform detailed statistical and trend analyses, quantifying publication growth, geographical distribution, and the evolution of research themes over time.

The bibliometric framework was designed to maintain strong alignment between the analytical process and the study′s objectives by incorporating four analytical dimensions. The first dimension, publication productivity, evaluated research output through annual publication trends, leading authors, institutions, and countries, providing a quantitative overview of global research activity. The second dimension, citation impact, measured scientific influence using total citations, h‐index, and key journals to identify the intellectual foundations and the most influential works shaping the field. The third dimension, collaboration networks, examined coauthorship patterns and institutional linkages, revealing the structure and intensity of international cooperation and interdisciplinary partnerships. The fourth dimension, keyword and thematic analysis, identified conceptual linkages, thematic clusters, and topic trend evolution through keyword co‐occurrence and thematic mapping, thereby tracing the development of research themes and technological convergence over time.

For the co‐occurrence analysis, thematic relevance was ensured by applying several filtering criteria to refine the selection of keywords. First, only those keywords that appeared with a minimum frequency threshold (e.g., ≥ 5) across the corpus were included in the analysis. To enhance consistency, synonyms and spelling variants, such as “IoT” and “Internet of Things,” were manually clustered and normalized. Additionally, irrelevant or overly generic terms, such as “human,” “male,” or “female,” were excluded through a process of manual screening. Additionally, to improve the thematic focus and domain specificity, co‐occurrence mapping was restricted to author keywords and indexed terms. These steps collectively ensured that the analysis captured relevant and meaningful relationships within the scope of smart packaging technologies.

These bibliometric insights map the technological convergence within the field and demonstrate how interdisciplinary research efforts and innovation trends are shaping the next generation of intelligent and sustainable packaging systems [26]. The bibliometric indicators outlined in Table 1 were selected to ensure direct alignment with the study′s objectives and analytical framework. Indicators related to publication productivity, citation impact, collaboration networks, and keyword co‐occurrence collectively provide a multidimensional understanding of the Smart Packaging 4.0 research landscape. Publication productivity captures the temporal and geographical distribution of scientific contributions, highlighting leading authors, institutions, and nations driving innovation. Citation impact metrics identify the most influential works and intellectual foundations that shape advancements in sensor integration, IoT connectivity, and traceability systems. Collaboration network analyses reveal the strength and diversity of interdisciplinary and international partnerships that accelerate knowledge diffusion and technological innovation. Meanwhile, keyword and thematic analyses uncover conceptual linkages, thematic clusters, and emerging research fronts, illustrating how technological themes evolve and converge toward sustainable and digitally integrated smart packaging ecosystems.

**Table 1:** Mapping of study objectives and corresponding bibliometric indicators.

Study objectives	Bibliometric indicators	Analytical contribution
To map the current research landscape and technological trends in smart packaging using bibliometric analysis tools such as VOSviewer and Bibliometrix (R Studio).	• Publication productivity indicators (annual publication growth, leading authors, institutions, and countries).• Keyword and thematic analysis (cluster mapping, thematic evolution, and overlay visualization).	Provides a structural overview of global research activity, showing temporal growth, leading contributors, and evolution of technological themes (e.g., biodegradable sensors and IoT packaging).
To analyze how technological advancements—particularly sensor integration and digital connectivity—improve product quality monitoring and traceability across the food supply chain.	• Citation impact indicators (most cited papers, total citations, h‐index, and influential journals).• Keyword co‐occurrence networks to identify dominant technologies (e.g., IoT, sensors, RFID, blockchain, traceability).• Cocitation analysis to trace intellectual linkages among foundational works.	Identifies the most influential studies, technologies, and concepts that define how digital tools and sensors enhance quality control and traceability in smart packaging systems.
To identify key knowledge gaps and opportunities for developing fully integrated and sustainable smart packaging systems.	• Thematic evolution and trend analysis (tracking emerging versus declining topics).• Collaboration network analysis (coauthorship and institutional linkages).• Thematic density maps to highlight underexplored areas.	Reveals emerging frontiers, research gaps, and collaboration potential, informing strategic directions for developing integrated, sustainable Smart Packaging 4.0 solutions.

### 2.3. Interpretations

Building upon the bibliometric analyses and the alignment framework presented in the previous section, the final phase centers on the interpretation and synthesis of the results to extract actionable insights that inform future research and innovation strategies. Thematic clusters identified through co‐occurrence, citation, and collaboration network analyses were critically examined to reveal emerging technologies, persistent knowledge gaps, and unresolved challenges within the Smart Packaging 4.0 domain. This interpretive phase transforms quantitative bibliometric patterns into a qualitative understanding of how research trajectories are evolving and where opportunities for technological convergence and integration are most pronounced.

The interpretive synthesis serves as a strategic decision‐support framework for researchers, policymakers, and industry stakeholders by highlighting priority innovation pathways, facilitating evidence‐based resource allocation, and addressing technical, economic, and regulatory barriers that constrain large‐scale adoption. Through this analytical lens, the bibliometric findings are converted into a strategic knowledge map, outlining how interdisciplinary advances in materials engineering, food science, and digital technologies can be leveraged to accelerate the development and implementation of next‐generation smart packaging systems aligned with Industry 4.0 principles.

## 3. Result

### 3.1. Research Landscape and Evolution of Smart Packaging Studies

#### 3.1.1. Annual Publication Trends

**Figure 2:**
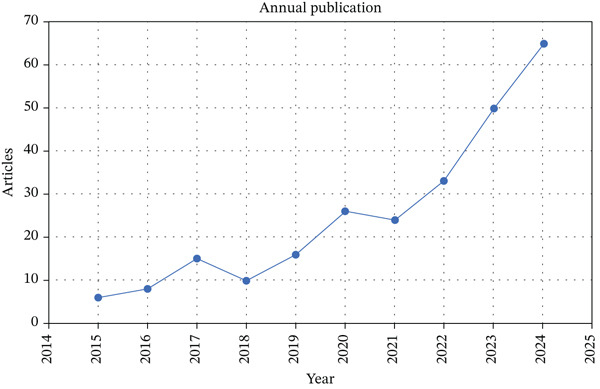
Annual publication trend on smart packaging research (2015–2024).

The bibliometric analysis reveals a marked growth in Smart Packaging 4.0 publications between 2015 and 2024 (Figure 2). Publication rates accelerated sharply after 2019, reflecting rising interest in sensor‐based food monitoring and digital traceability technologies. Key advances include studies on biosensors and smart packaging films that enable real‐time monitoring of microbial contamination, chemical hazards, and freshness, thereby enhancing food safety and traceability [13, 27]. Additionally, the development of biodegradable, bio‐based materials—such as smart chitosan films embedded with sensory active compounds—enables continuous monitoring across the supply chain via real‐time sensing and data transmission [28].

This upward trend reflects the increasing demand for sustainable and efficient food safety solutions. Research is increasingly focused on integrating sensors to optimize supply chains and enhance traceability. The convergence of sensor technologies, communication technologies, data analytics, and eco‐friendly materials is transforming the field, making it more interdisciplinary. As the volume of publications grows, the smart packaging industry is progressing from experimental models to scalable applications. However, challenges such as scalability and regulatory hurdles persist. The expanding body of literature lays a solid foundation for future innovations in this area.

**Figure 3:**
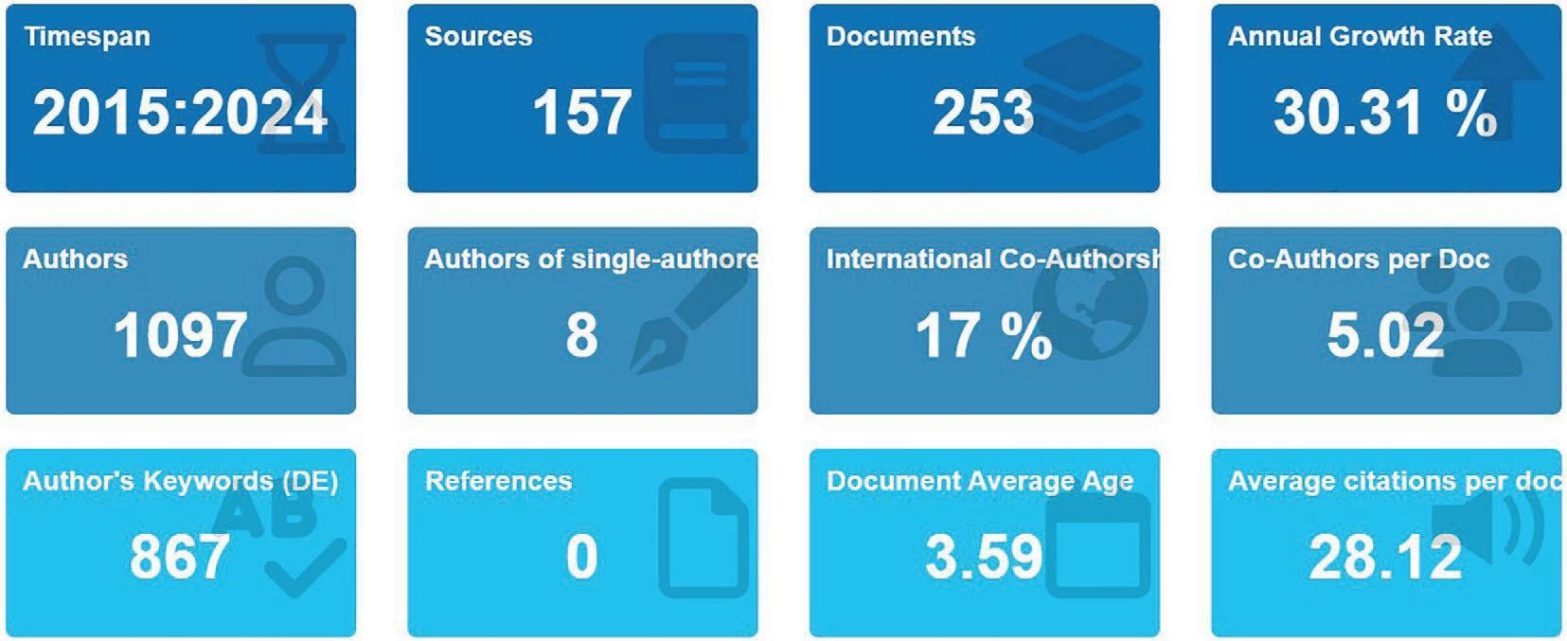
Descriptive bibliometric indicators of smart packaging literature (2015–2024).

As summarized in Figure 3, a total of 253 documents from 157 publications, authored by 1097 researchers with an average of 5.02 coauthors per paper and 17% international collaborations, illustrate a highly collaborative and interdisciplinary research environment driving innovations in smart packaging. This global cooperation reflects the convergence of food science, material engineering, and digital technology necessary for advancing intelligent packaging solutions.

#### 3.1.2. Most Cited Papers

Table 2 provides insights into influential research themes in the field of smart food packaging, with a focus on pH‐sensitive, biodegradable, and intelligent packaging technologies. These themes underscore the integration of natural materials, such as chitosan, gelatin, and anthocyanins, into packaging systems to monitor food freshness and quality. A prominent feature in many of the studies listed is the pH sensitivity of the films, enabling real‐time freshness monitoring by detecting changes in food′s pH or spoilage indicators, such as ammonia release. This aligns with the growing need for more sustainable and interactive packaging solutions in food safety and preservation.

**Table 2:** The most cited papers and their influential research themes in smart food packaging.

Paper	Citations	Research theme
[29]	278	Smart packaging made from chitosan, corn starch, and red cabbage extract, designed with a pH‐sensitive indicator for detecting fish spoilage.
[30]	248	Smart edible films incorporating gelatin and curcumin, which change color in response to pH levels, offering a visual indicator for food quality.
[31]	239	PH‐responsive films with barberry anthocyanins and methylcellulose/chitosan nanofibers, providing real‐time freshness monitoring of meat and seafood.
[32]	195	PH‐sensitive, biodegradable smart packaging films featuring red barberry anthocyanins and chitin nanofibers, enabling freshness monitoring and spoilage detection.
[33]	180	High‐strength, hydrophobic colorimetric sensing films made from biodegradable cellulose and naphthoquinone dyes, suitable for shrimp and pork freshness monitoring.
[34]	155	Curcumin‐loaded chitosan/PEO nanofiber films for intelligent packaging, enabling real‐time monitoring of chicken freshness with visible color changes.
[35]	147	Active films made from cassava starch and Chinese bayberry anthocyanins, improving mechanical properties for pork freshness monitoring through pH sensitivity.
[36]	139	Gelatin‐based smart edible films made with red cabbage extracts, combining antioxidant properties with pH‐sensitive color changes for active food packaging.
[37]	133	Biodegradable pH‐sensitive films using jambolão skins extract in methylcellulose, offering antioxidant properties and environmental benefits for food packaging.
[38]	119	Gelatin‐based packaging films incorporating lavender essential oil emulsions and Alizarin, providing antibacterial and real‐time freshness monitoring for shrimp.

Several studies explore the integration of natural plant‐based extracts—such as red cabbage, Chinese bayberry, and jambolão skins—into biodegradable films, offering both antioxidant and pH‐responsive properties. For instance, Silva‐Pereira et al. and Musso et al. introduced edible films incorporating red cabbage extract and curcumin, respectively, which changed color in response to pH fluctuations, providing a visual indication of food spoilage [29, 30]. These developments enhance consumer engagement by offering an easy‐to‐understand visual cue of freshness, an essential aspect of intelligent packaging.

The most cited papers demonstrate a strong interdisciplinary foundation, highlighting how smart packaging research integrates innovations in food science, materials engineering, and digital technologies to enhance food quality, safety, and sustainability. From a food science perspective, most of the highly cited studies focus on freshness monitoring, spoilage detection, and quality assurance, addressing critical challenges in food preservation. Research exemplifies the development of edible, pH‐sensitive films that visually indicate freshness through color changes, allowing consumers to assess product quality in real time [29, 30]. These contributions reflect the evolution of food packaging from passive containment systems to interactive platforms capable of responding to biochemical changes during storage and distribution.

At the same time, the materials engineering dimension is prominently represented through the extensive use of natural biopolymers (e.g., chitosan, gelatin, starch, and cellulose) and plant‐based extracts (e.g., red cabbage, bayberry, and jambolão skins) that impart both functional and environmental benefits. Studies highlight the development of biodegradable and mechanically robust films, emphasizing sustainability while maintaining essential protective properties such as tensile strength and moisture resistance [32, 37]. The recurring theme of biodegradability underscores the growing commitment to eco‐friendly and renewable materials, aligning smart packaging research with global sustainability objectives and the circular economy principles of Industry 4.0.

As these technologies advance, the integration of sensor and IoT systems has emerged as a defining trend that connects physical packaging materials with digital intelligence. This convergence is illustrated through packaging films embedded with sensing elements capable of detecting environmental changes such as temperature, humidity, and spoilage gasses [31, 34]. These smart materials not only respond to food quality variations but also communicate this information through digital interfaces, enabling real‐time monitoring and data‐driven decision‐making across the supply chain. Such innovations lay the groundwork for IoT‐enabled smart packaging systems that enhance traceability, automate quality control, and optimize logistics operations, illustrating the ongoing technological convergence that defines the Smart Packaging 4.0 landscape—where food science ensures safety and functionality, materials engineering provides sustainable and responsive substrates, and digital technologies enable intelligent connectivity.

#### 3.1.3. Most Influential Sources

**Figure 4:**
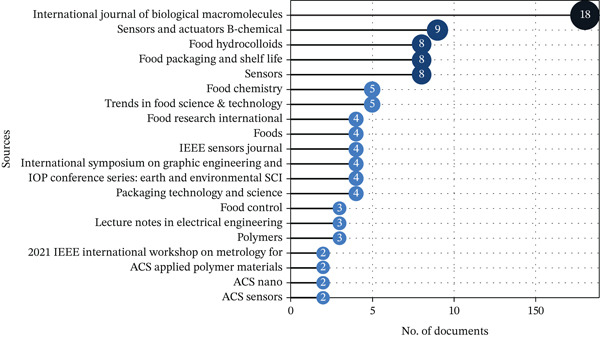
Distribution of publications across top journals, illustrating the interdisciplinary scope of smart packaging research across food, materials, and sensor technologies.

The distribution of publications across journals (Figure 4) demonstrates the interdisciplinary foundation of smart packaging research, spanning food science, materials engineering, and digital technologies. The International Journal of Biological Macromolecules (18 documents) dominates the dataset, emphasizing research on biopolymer and biodegradable materials essential for sustainable packaging solutions. Likewise, journals such as Food Hydrocolloids, Food Packaging and Shelf Life, and Food Chemistry highlight the food science dimension, where innovations in preservation, quality control, and safety monitoring are central to ensuring product integrity. These sources collectively indicate a strong materials–food nexus, reflecting efforts to merge biopolymer innovation with practical applications in food packaging.

In parallel, technology‐oriented journals such as Sensors and Actuators B: Chemical, Sensors, and IEEE Sensors Journal illustrate the integration of sensor systems, IoT connectivity, and real‐time monitoring technologies—core components of Industry 4.0‐enabled smart packaging. Complementary sources like Polymers, ACS Applied Polymer Materials, and ACS Nano further capture the nanotechnology and materials engineering aspects that enable responsive and intelligent packaging functionalities. The diversity of these publication outlets confirms that the dataset effectively represents the technological convergence driving Smart Packaging 4.0, where food science, digital innovation, and sustainable materials research coalesce to form an integrated, data‐driven, and eco‐efficient packaging ecosystem.

#### 3.1.4. Most Influential Countries

**Figure 5:**
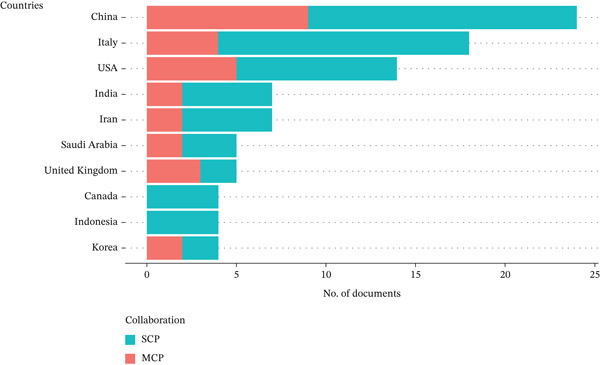
Corresponding author′s countries concerning smart packaging.

The analysis of corresponding authors′ countries reveals distinct patterns of national research leadership and international collaboration (Figure 5). China emerges as the dominant contributor with 24 publications, suggesting a strong institutional infrastructure and growing industrial demand driving innovation in smart packaging. Notably, 62.5% of China′s output comprises single‐country publications (SCP), reflecting a primarily domestic research focus, likely supported by targeted national funding and policy initiatives. Significantly, the most influential and highly cited studies from China in this domain also fall under the SCP category, underscoring the strength and impact of internally driven research efforts. These studies, which have advanced areas such as colorimetric FIs and biodegradable packaging materials, have achieved wide recognition without relying on international collaboration, pointing to a self‐sustaining research ecosystem. Italy and the United States follow closely, with Italy showing a high SCP ratio (77.8%), indicative of a consolidated internal network with less reliance on external partners. The United States, in contrast, balances domestic and collaborative work (35.7% MCP), highlighting its position as both a leader and a connector in global food packaging research. Both countries′ patterns reflect strategic alignment with their national priorities—Italy in biosensor technologies and the United States in active intelligent packaging systems. The prominence of SCPs among top‐cited works across several leading countries suggests that domestic research capacity remains a cornerstone of impactful innovation in the smart packaging field.

In contrast, countries such as the United Kingdom and Korea exhibit significantly higher international collaboration ratios (MCP 60% and 50%, respectively), suggesting a strategic orientation toward cross‐border partnerships, possibly influenced by funding ecosystems like Horizon Europe and bilateral R&D agreements. These countries may not lead in volume but serve as critical collaborators in advancing interdisciplinary innovations, such as developing disposable wireless sensors and curcumin for detecting food spoilage [39, 40]. Meanwhile, nations like Saudi Arabia and Iran maintain moderate MCP levels (40% and 28.6%), pointing to evolving but increasingly outward‐looking research strategies. Interestingly, Canada and Indonesia reported no multiple‐country publications, focusing on sensors for real‐time monitoring, implying localized research endeavors that may benefit from greater global engagement [41]. The overall landscape shows that while volume leaders (e.g., China and Italy) sustain innovation through strong domestic frameworks, countries with higher MCP proportions are shaping a more integrated global research ecosystem essential for addressing complex, transnational challenges in food safety and sustainable packaging technologies.

### 3.2. Trends and Thematic Analysis

#### 3.2.1. Research Trends and Technological Phases (2015–2024)

**Figure 6:**
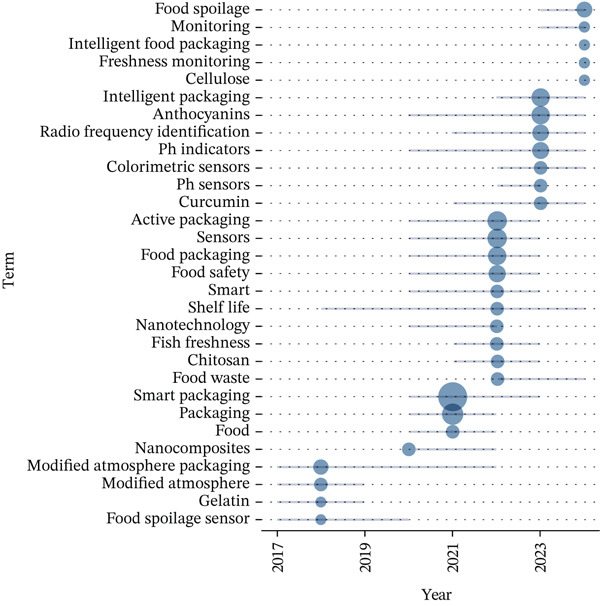
The research trends in smart packaging have evolved significantly over the past several years.

Significant advancements in smart packaging technologies over the past several years have transformed the landscape of food packaging systems. These innovations are closely linked to developments in materials science and the increasing integration of digital technologies. The bibliometric analysis, as shown in Figure 6, identifies key technological trends that have emerged between 2015 and 2024, reflecting a shift toward more sustainable, intelligent, and data‐driven packaging solutions.

From 2015 to 2019, the focus was on enhancing food preservation through modified atmosphere packaging (MAP) and food spoilage sensors. During this period, the development of colorimetric sensors and the use of gelatin‐based films marked the early integration of environmental monitoring and food safety technologies into packaging materials. The period between 2020 and 2022 saw more advanced technologies emerge, such as the use of biodegradable materials and anthocyanins or pH‐responsive films for real‐time freshness monitoring. By 2023–2024, the integration of IoT, artificial intelligence (AI), and blockchain technologies marked the peak of smart packaging innovation, leading to the concept of Smart Packaging 4.0. This new phase emphasizes digital traceability and predictive monitoring, marking a significant shift toward data‐driven and intelligent packaging systems designed for greater efficiency, sustainability, and food safety. The analysis outlines three distinct phases in the evolution of smart packaging research, as summarized in Table 3.

**Table 3:** Summary of technological phases of smart packaging research (2015–2024).

Phase/period	Core research focus	Dominant materials and functional components	Digital/sensor technologies	Primary research goals	Representative studies
Phase I: Foundation of smart packaging (2017–2019)	Modified atmosphere packaging (MAP) and early colorimetric spoilage indicators	‐ Biopolymers (chitosan, starch, and cellulose)‐ Natural pigments (anthocyanins and curcumin)‐ Gas‐sensitive films	Limited digital integration; colorimetric and pH‐sensitive indicators	‐ Visual monitoring of freshness‐ Detection of spoilage gasses (CO_2_, NH_3_)‐ Extending shelf life	[29, 30, 42]
Phase II: biodegradable and functionalized materials (2020–2022)	Biodegradable active packaging with sensor embedding and RFID integration	‐ Polylactic acid (PLA), gelatin, and cellulose‐based nanocomposites‐ Metallic nanoparticles (ZnO and AgNPs) for antimicrobial functions	‐ RFID tags and basic wireless data logging‐ Early adoption of printed biosensors	‐ Combine sustainability and smart sensing‐ Real‐time data acquisition of food quality‐ Reduce environmental impact	[31, 33, 43]
Phase III: Smart Packaging 4.0 (2023–2024)	Integration of IoT, AI, and blockchain technologies for real‐time predictive monitoring	‐ Biodegradable polymers with embedded flexible electronics‐ Hybrid nanomaterials for multisensing	‐ IoT networks, NFC communication, and cloud platforms‐ AI‐driven data analytics and blockchain traceability	‐ Intelligent decision systems‐ Predictive shelf‐life estimation‐ Transparent and secure food traceability	[12, 44, 45]

#### 3.2.2. Co‐occurrence Analysis and Thematic Clusters

**Figure 7:**
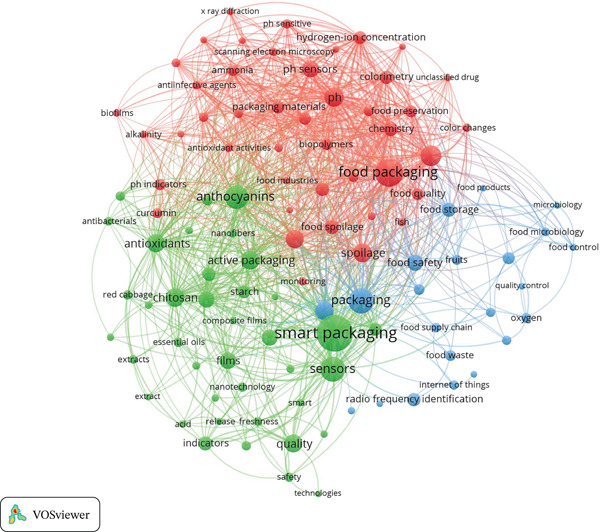
The VOSviewer co‐occurrence analysis visualization illustrates three thematic clusters.

To understand what the research themes in smart packaging and their relationships with sensors and digital technologies in smart food packaging systems, we conducted a co‐occurrence analysis using VOSviewer software. We used “all keywords” as the unit of analysis, “full count” as the counting method, and selected keywords with at least five occurrences (*n* = 5). This resulted in 105 keywords. The results presented in Figure 7 illustrate the relationships between keywords in the context of smart packaging research. A summary of the keywords and their main themes is provided in Table 4.

**Table 4:** Summary of thematic clusters from co‐occurrences analysis with VOSViewer.

Cluster	Main keywords	Themes
Cluster 1 (red color)	Food packaging (55), color (32), pH (29), spoilage (27), intelligent packaging (26), ph sensors (23), chemistry, food spoilage, colorimetry, and food quality	Developing chemical indicators, such as pH sensors and colorimetric indicators, to detect spoilage in food through physicochemical changes like pH variations or ammonia presence, enhancing food safety and quality in real‐time smart packaging systems.
Cluster 2 (green color)	Smart packaging (85), sensors (44), anthocyanins (40), active packaging (28), chitosan (28), antioxidants (26), quality (26), films (23), nanocomposites, indicators, nanoparticles, and starch	Focusing on the use of natural substances like anthocyanins and chitosan, this cluster integrates them into active packaging materials to improve food quality, extend freshness, and offer sustainability. Combining these compounds with smart sensors and nanocomposites enhances preservation and shelf life.
Cluster 3 (blue color)	Packaging (45), shelf life (28), food safety (22), food storage (17), RFID (16), MAP (14), food waste (11), temperature (11), IoT (9)	Highlighting the application of digital technologies like RFID and IoT, this cluster focuses on monitoring logistics conditions to ensure food safety. Integrating these technologies with packaging systems like MAP extends shelf life, improves quality, and enhances supply chain efficiency while minimizing waste.

The red cluster emphasizes the integration of sensor technologies into food packaging, particularly focusing on the monitoring of food quality. The keyword “food packaging” (55 occurrences) forms the core of this cluster, indicating the widespread interest in how packaging materials can be optimized for food safety and preservation. pH sensors (23 occurrences) and colorimetry (32 occurrences) are essential components within this cluster, offering real‐time detection of spoilage by measuring changes in acidity or color. These sensors have been applied to detect spoilage by tracking the acidity or alkalinity of food [33, 46], whereas other studies examined the use of colorimetric sensors to detect subtle color changes indicative of food deterioration [32].

The presence of food spoilage (27 occurrences) and food quality (related terms such as chemistry) highlights the key role these technologies play in improving food preservation. Packaging systems embedded with these sensors provide immediate feedback on freshness, empowering both consumers and suppliers to act quickly. Moreover, the integration of biodegradable packaging materials alongside these sensors is a key development in reducing food waste. The use of biodegradable materials helps mitigate the environmental impacts of packaging waste while ensuring that only safe and fresh food reaches consumers [47–49].

Additionally, this cluster emphasizes the concept of intelligent packaging, which utilizes sensors to monitor variables such as temperature, humidity, and spoilage indicators in real‐time. These technologies enable more informed decisions about food safety and freshness, which is crucial for reducing food waste and improving food safety [13, 50]. The integration of biopolymers further strengthens packaging integrity, responding to external factors that accelerate food deterioration [51, 52].

The green cluster delves into the evolution of smart packaging (85 occurrences) and its transformative potential through the integration of advanced sensor technologies (44 occurrences) and active packaging (28 occurrences). Unlike traditional packaging, smart packaging systems are designed to monitor real‐time changes in environmental factors such as temperature, humidity, and gas composition and facilitate immediate corrective actions to preserve food quality. Smart packaging systems not only monitor changes but also adjust the internal environment of the package to optimize food storage, thus extending shelf life [13, 24, 53].

Active packaging systems go beyond passive monitoring by dynamically adjusting the atmosphere within the packaging, such as by absorbing or releasing gasses like oxygen and carbon dioxide, to slow spoilage and preserve food integrity [23, 54]. A particularly innovative aspect of this cluster is the use of anthocyanins (40 occurrences), natural pigments that change color in response to pH fluctuations, providing an immediate and visual indicator of food freshness and spoilage [48, 55].

Additionally, chitosan (28 occurrences) and antioxidants (26 occurrences) are incorporated into packaging materials, providing antimicrobial and antioxidant properties that further enhance food preservation and safety [27, 56]. The use of nanocomposites and films (23 occurrences) enables the development of multifunctional packaging systems that combine both preservation and spoilage detection in one solution [57]. This cluster, therefore, offers a holistic approach to food preservation by combining innovative sensor technologies, active packaging, and bioactive materials, all while addressing sustainability concerns in food packaging.

The blue cluster focuses on the integration of digital technologies within food supply chains to enhance efficiency, safety, and traceability. RFID (16 occurrences) and IoT (9 occurrences) are central to this cluster, enabling precise tracking of food products throughout the supply chain. These technologies enable producers, suppliers, and distributors to monitor the condition and location of products at each stage of the supply chain, ensuring optimal food safety [12, 45, 58].

They also track environmental factors like temperature (11 occurrences) and humidity, minimizing the risks of contamination and spoilage. Additionally, packaging systems such as MAP (14 occurrences) play a crucial role in preserving food quality by adjusting gas composition to slow food deterioration [21, 59, 60].

The integration of smart packaging with digital tracking technologies also facilitates more efficient logistics management, reducing overstocking, spoilage, and waste. Real‐time data from these systems optimize inventory management and reduce food waste, contributing to a more sustainable supply chain [61, 62]. The combination of RFID and IoT technologies further streamlines the distribution process, minimizing the environmental footprint and ensuring food safety from production to consumption [14, 18].

This cluster represents a significant shift toward a more connected and efficient food supply chain that uses technology to enhance safety, reduce waste, and improve operational sustainability.

## 4. Discussion

This study highlights the development of Smart Packaging 4.0, marked by the rapid technological convergence of materials engineering, food science, and digital connectivity, as evidenced by the thematic clusters identified in the bibliometric analysis. The discussion critically interprets these quantitative trends, transforming the research landscape into a strategic synthesis of the field′s evolution and its future direction. The analysis delves into the role of innovations in sustainable materials, advanced sensing technologies, and digital integration, such as IoT, AI, and blockchain, in shaping the core of Smart Packaging 4.0 systems. Additionally, the influence of global regulatory frameworks and sustainability commitments is examined, considering how these advancements align with international standards and the principles of the circular economy. The market dynamics surrounding consumer acceptance are explored, emphasizing the growing demand for transparent, real‐time information and the importance of institutional trust in facilitating widespread adoption. This analysis is aimed at identifying key innovation pathways and addressing the technical, economic, and regulatory challenges that hinder large‐scale implementation.

### 4.1. Advancements in Smart Packaging Technologies

Smart Packaging represents a crucial advancement that integrates the functions of Intelligent Packaging, focused on monitoring and communication, and active packaging, dedicated to preservation and shelf‐life extension. This technological convergence creates a cyber‐physical system designed to enhance food safety, improve supply chain efficiency, and mitigate global food waste issues [19].

#### 4.1.1. Material and Sensor Innovation

The fundamental transformation of food packaging into the Smart Packaging 4.0 paradigm is anchored in the convergence of material science and advanced sensing technologies. This evolution is primarily characterized by a transition from passive, petroleum‐based barriers to active and intelligent interfaces capable of real‐time monitoring and preservation. An in‐depth analysis of these components reveals an intricate interplay between structural polymer dynamics, nanoscale reinforcements, and the integration of digital intelligence [27].

##### 4.1.1.1. Sustainable Biopolymer Matrices: Structural and Functional Trade‐Offs.

The selection of biopolymers as the primary matrix for smart packaging is largely driven by the urgent need to replace nonbiodegradable, petroleum‐based plastics such as polyethylene terephthalate (PET), polypropylene (PP), and polystyrene (PS), which are major contributors to global microplastic pollution [5]. Despite their promise as sustainable alternatives, a closer technical analysis of these so‐called “green” materials reveals important trade‐offs between their environmental advantages and functional performance [9].

Among polysaccharide‐based biopolymers, chitosan and starch are two of the most widely studied candidates. Chitosan stands out due to its inherent antimicrobial and metal‐chelating properties, which arise from the positive charges in its polycationic structure that enable disruption of microbial cell membranes. This makes it particularly suitable for food packaging applications that demand active protection against microbial contamination. In contrast, starch is recognized for its abundance, renewability, and low cost, positioning it as an economically attractive option. However, its high hygroscopicity and limited mechanical strength pose serious drawbacks, as it tends to absorb moisture and lose structural integrity in high‐humidity environments typical of fresh produce or meat packaging [5].

Microbial polyesters such as polylactic acid (PLA) and polyhydroxyalkanoates (PHA) have emerged as another promising class of biopolymers for smart packaging. These materials exhibit thermal and mechanical stability that can rival conventional polyolefins, making them viable candidates for industrial processing and commercial‐scale applications. PLA, in particular, is valued for its compatibility with extrusion and molding techniques, offering good rigidity and transparency. However, its inherent brittleness limits its flexibility, often necessitating the incorporation of plasticizers or blending with other polymers to tailor its mechanical performance for film‐based packaging applications [63].

##### 4.1.1.2. Nanocomposites: Mechanisms of Functional Reinforcement.

To bridge the performance gap between pure biopolymers and conventional synthetic plastics, the incorporation of nanofillers into polymer matrices has emerged as a cornerstone of smart material innovation. The addition of nanoscale materials enhances the mechanical, barrier, and functional properties of biopolymers, enabling them to better meet the demands of modern packaging applications [63].

According to the tortuous path model, the inclusion of nanoscale additives such as cellulose nanocrystals (CNCs), cellulose nanofibrils (CNFs), or nanoclays (e.g., montmorillonite, MMT) significantly improves gas and moisture barrier performance. These high‐aspect‐ratio fillers create a more complex diffusion pathway that forces penetrant molecules, such as oxygen or water vapor, to travel along an elongated and irregular route through the polymer matrix. This effectively reduces permeability and enhances the shelf life of packaged products by impeding the transmission of gasses and moisture [5].

Metallic and metal oxide nanoparticles, particularly zinc oxide (ZnO) and titanium dioxide (TiO_2_), play multifunctional roles in enhancing smart packaging performance. In addition to providing structural reinforcement, these nanoparticles contribute UV‐shielding capabilities and exhibit broad‐spectrum antimicrobial activity. Their antimicrobial mechanisms are primarily attributed to the generation of reactive oxygen species (ROS) or the controlled release of metal ions, both of which disrupt microbial cell membranes and inhibit the proliferation of foodborne pathogens. However, although these nanoparticles offer distinct functional advantages, excessive loading can lead to undesirable particle agglomeration. This aggregation introduces stress concentration points within the polymer matrix, which compromises mechanical integrity and reduces the overall performance of the material [63].

##### 4.1.1.3. Dynamics of Freshness and pH Indicators.

FIs function as visual communication tools that detect biochemical markers of food spoilage, particularly total volatile basic nitrogen (TVB‐N) compounds, which accumulate as proteins degrade in perishable foods like meat and seafood [64]. These smart indicators translate chemical changes into visible color shifts, allowing consumers and retailers to assess product freshness in real time without opening the package.

The halochromic response of natural pigments has gained significant attention as a safer and more sustainable alternative to synthetic dyes, aligning with clean‐label and consumer safety trends. Natural colorants such as anthocyanins and curcumin are widely studied for this purpose. Anthocyanins exhibit a distinct color transition caused by structural transformations in response to pH variations—shifting from a red flavylium cation form under acidic conditions to blue or yellow quinoidal and chalcone structures in alkaline environments. This property enables anthocyanin‐based indicators to provide an intuitive visual signal corresponding to the rise in basic spoilage compounds during food deterioration, particularly in meat and seafood packaging [31].

Despite their high sensitivity, anthocyanins face notable limitations in stability. They are prone to photodegradation and thermal breakdown, which can compromise their reliability during processing and storage. Comparative studies show that curcumin demonstrates greater thermal stability under high‐temperature conditions such as extrusion, making it advantageous for industrial applications. However, its pH‐dependent color range is narrower than that of anthocyanins. To overcome these constraints, researchers are developing stabilization strategies including copigmentation with tannic acid and encapsulation within nanoparticles to enhance the pigments′ resistance to light, oxygen, and heat exposure [40].

##### 4.1.1.4. Advanced Fabrication and Digital Integration.

The transition of smart packaging from laboratory prototypes to scalable commercial solutions relies heavily on advances in fabrication technologies and the incorporation of digital connectivity into packaging systems. These developments are reshaping the packaging landscape, enabling multifunctional materials that not only protect products but also sense, respond, and communicate in real time [65].

High‐resolution manufacturing techniques are at the forefront of this transformation. Traditional solvent casting methods are increasingly being replaced by electrospinning, which enables the production of nanofibrous membranes (NFMs) with exceptionally high surface‐to‐volume ratios. This structural advantage allows NFMs to interact almost instantaneously with gaseous biomarkers, dramatically accelerating sensing responses compared with conventional films [66]. In parallel, additive manufacturing methods such as 3D printing and inkjet printing have emerged as powerful tools for fabricating smart packaging systems. These technologies allow for the precise, scalable deposition of functional materials, including responsive polymers, pigments, and even flexible or edible electronics, directly onto packaging substrates—paving the way for mass customization and efficient large‐scale production [65].

The next phase of advancement, often referred to as Smart Packaging 4.0, centers on the integration of material‐based sensors with cyber‐physical systems. This involves embedding IoT, RFID, and near‐field communication (NFC) technologies into packaging to transform physical and chemical sensor signals—such as pH, humidity, or temperature—into digital data. Such interconnected systems enable autonomous product monitoring, real‐time traceability, and data‐driven logistics strategies like first‐expired‐first‐out (FEFO). Through these innovations, the package evolves into an intelligent data carrier capable of continuously communicating food quality and safety information across the global supply chain [20].

#### 4.1.2. Digital Convergence and Connectivity

The evolution toward Smart Packaging 4.0 represents a paradigm shift where the package is transformed from a passive container into an intelligent communicator within a cyber‐physical system. This transformation is facilitated by the convergence of various digital technologies that enable the seamless flow of information between materials, stakeholders, and data processing platforms [8].

##### 4.1.2.1. Data Acquisition: IoT, RFID, and Wireless Sensor Networks (WSN).

The integration of IoT serves as the foundational infrastructure, extending network connectivity and computing capability to everyday packaging objects [61, 67]. Unlike traditional identification systems, RFID technology provides item‐level granularity and nonline‐of‐sight data collection, allowing for the simultaneous tracking of hundreds of items [68].

An in‐depth analysis reveals that this connectivity does not merely record location; it facilitates a dynamic data stream concerning critical environmental parameters such as temperature, humidity, and gas concentrations. By integrating WSN, the system creates a hierarchical network where tags coordinated by master nodes integrate physical parameters into global enterprise information systems. This transition enables the shift from a rigid first‐in‐first‐out (FIFO) inventory model to a data‐driven FEFO strategy, which is critical for reducing food waste in perishable goods supply chains [11].

##### 4.1.2.2. Interactive Consumer Interfaces: NFC and Digital Engagement.

NFC technology has emerged as a specialized subset of RFID that prioritizes consumer‐facing communication and user interaction [24]. Because many modern smartphones embed integrated NFC readers, consumers can immediately access real‐time quality data, provenance, and nutritional information by simply tapping the package [13].

However, behavioral analysis suggests that the effectiveness of these interfaces is contingent upon user interface (UI) design and market demographics. Research using eye‐tracking technology indicates that NFC tags positioned in the “center‐right” of the packaging with clear activation prompts achieve the highest visual attention and engagement. Furthermore, generational analysis shows that although Y and Z generations are familiar with these technologies, broader adoption requires simplifying the user experience through intuitive apps and visual indicators that convert complex sensor data into easy‐to‐understand freshness cues [69].

##### 4.1.2.3. Data Integrity: Blockchain Technology.

The massive data streams generated by IoT‐enabled packaging require robust protocols to ensure trust and security [11, 67]. Blockchain technology addresses this by providing a decentralized, immutable ledger that permanently records every transaction and quality check along the supply chain [50].

The analytical significance of blockchain lies in its ability to resolve information asymmetry and the “trust gap” in global food trade [9]. By coupling blockchain with RFID/NFC, stakeholders can verify that environmental conditions were maintained without fear of data manipulation by malicious third parties [67]. This creates a tamper‐proof digital identity for each food product, facilitating targeted recalls and enhancing consumer confidence in the authenticity of high‐value commodities [50].

##### 4.1.2.4. Computational Intelligence: AI and Machine Learning (ML).

As the number of connected objects grows, systems must manage big data—potentially reaching terabytes daily—making advanced analytical tools indispensable [68]. AI and ML algorithms are employed to analyze these complex, often heterogeneous datasets to provide reliable predictions for food quality [70].

In‐depth analysis shows that deep learning (DL), particularly long short‐term memory (LSTM) networks, are exceptionally effective for analyzing multidimensional time‐series data from sensors to predict spoilage and remaining shelf life(RSL) with high precision. For instance, ML models have demonstrated over 95% accuracy in classifying the freshness of protein‐rich foods by processing signals from gas and temperature sensors [61]. The emergence of TinyML further advances this by allowing these complex models to run locally on low‐power microcontrollers at the “edge,” reducing the latency and energy costs associated with cloud computing while ensuring real‐time responsiveness [71].

#### 4.1.3. The Role of Actuators: Toward Autonomous Responsive Systems (The Execution Unit)

The evolution toward Smart Packaging 4.0 is defined by the integration of actuators, which transform the package from a simple monitoring tool into an autonomous execution unit. Within this cyber‐physical framework, actuators serve as the “muscles” of the system, closing the loop between data acquisition and physical intervention to maintain food quality and safety [8, 20].

##### 4.1.3.1. Transition From Informative to Corrective Systems.

Traditional intelligent packaging is primarily informative, relying on sensors and indicators to detect and communicate product status (e.g., pH changes or temperature abuse) to users. However, these systems are inherently reactive and cannot remediate quality loss without human intervention. The transition to corrective responsive packaging marks a significant advancement where actuators take physical action—such as releasing active agents or scavenging undesirable molecules—based on environmental triggers. This shift enables a “predict‐and‐prevent” model where the system senses an internal or external stimulus and executes a remediation protocol in real‐time, effectively extending the product′s shelf life beyond what is possible with passive barriers. By automating the preservation process, corrective systems mitigate the risks associated with “false negatives” in informative packaging, where spoilage might be detected too late for intervention [20].

##### 4.1.3.2. Controlled Release Mechanism in Active Packaging.

Active packaging functionality is optimized through on demand controlled release mechanisms, where chemical or physical actuators trigger the emission of antimicrobials, antioxidants, or scavengers only when specific thresholds are breached. Unlike traditional active systems that release agents continuously—potentially leading to the “dumping effect” and premature loss of packaging function—corrective actuators respond to precise biomarkers. For example, bacterial protease secreted during the growth of *B. cereus* can act as a trigger to degrade proteoliposomes, releasing cinnamon essential oil only when contamination is present. Similarly, moisture‐responsive actuators can initiate the generation of chlorine dioxide (ClO_2_) gas through the interaction of citric acid and sodium chloride, providing broad‐spectrum protection in response to high humidity or product respiration. These systems allow for a proportionate response where the concentration of active agents aligns with the actual degree of environmental threat, thereby enhancing safety while minimizing chemical migration into the food [20, 72].

##### 4.1.3.3. Innovation in Edible Robotics and Smart Materials.

The convergence of soft robotics and material science has facilitated the development of biopolymer‐based actuators that provide a tangible, mechanical interface for food safety [65]. These smart materials, often composed of chitosan, gelatin, or alginate, can undergo programmed shape transformations—such as bending, shrinking, or swelling—as a direct physical response to changes in pH, moisture, or temperature. In contrast to digital alerts that require a smartphone reader, these soft actuators serve as intuitive visual warnings for consumers; for instance, a label might physically “contract” or change its orientation if the cold chain is broken [73]. Furthermore, advances in edible robotics allow for the integration of these mechanical components with edible electronics, creating ingestible systems that can perform diagnostic or corrective tasks within the gastrointestinal tract [65]. This synergy between bio‐based materials and autonomous actuation not only enhances the functional reliability of smart packaging but also aligns with circular economy principles by ensuring that the entire “execution unit” remains biodegradable and safe for the environment [66, 74].

#### 4.1.4. Operational Supply Chain and Consumer Interface

The convergence of material and digital innovations within the Smart Packaging 4.0 framework fundamentally redefines the operational dynamics of the food supply chain. This interface serves as the critical link where raw sensor data are translated into logistical actions and consumer‐facing transparency, facilitating a transition from traditional linear models to highly responsive, cyber‐physical ecosystems [75].

##### 4.1.4.1. Supply Chain Resilience (SCRes) and Multinode Synchronization.

The integration of IoT and RFID technologies across supply chain nodes—from manufacturers and processors to distributors and retailers—enables unprecedented levels of end‐to‐end visibility [62]. An in‐depth analysis suggests that this connectivity is a primary driver of SCRes. By providing a continuous data stream of environmental parameters, these technologies allow the supply chain to transition from a reactive “detect‐and‐fix” approach to a proactive “predict‐and‐prevent” model [9]. This synchronization ensures that any deviation in the cold chain or product integrity is immediately identified, allowing for autonomous rerouting or targeted recalls that mitigate the “ripple effect” of disruptions [62]. Consequently, the package functions not merely as a container but as a traceable resource unit (TRU) that maintains a digital identity, ensuring data integrity through distributed ledgers like blockchain to resolve the “trust gap” in global food trade [9].

##### 4.1.4.2. Dynamic Inventory Management: The Transition to FEFO Models.

Traditional inventory management has historically relied on the FIFO method, which often results in significant food waste due to its reliance on static expiration dates that do not account for actual storage conditions. Smart packaging enables a paradigm shift toward FEFO strategies [11]. This transition is powered by time–temperature indicators (TTIs) and gas sensors that provide real‐time estimates of RSL [66]. Analytical modeling using AI and ML algorithms—such as LSTM networks—can process heterogeneous sensor data to predict spoilage with high precision [76]. By ranking items according to their dynamic freshness status rather than arbitrary dates, retailers can optimize stock rotation and implement dynamic pricing, effectively reducing the 30% of global food waste typically lost during the distribution and retail phases [9].

##### 4.1.4.3. Digital Consumer Engagement and Mitigation of Information Asymmetry.

Consumer‐facing technologies, particularly NFC and quick response (QR) codes, represent a transformative UI that bridges the gap between the product and the end user [69]. These interfaces address information asymmetry, where the consumer lacks detailed knowledge of a product′s provenance or actual quality [77]. In‐depth behavioral analysis using eye‐tracking technology indicates that the effectiveness of these interfaces depends on strategic design; elements placed in the “center‐right” of the package with clear activation prompts achieve the highest engagement. Beyond simple identification, these digital tags empower consumers with “digital material passports,” providing instant access to nutritional profiles, allergen alerts, and sustainability metrics [40]. This level of transparency not only enhances consumer confidence but also fosters a value cocreation environment where the consumer is actively involved in the quality assurance process, increasing brand loyalty and supporting the goals of a circular economy [78].

##### 4.1.4.4. Cold Chain Integrity and Automated Quality Redesign.

The “last mile” of the cold chain remains the most vulnerable segment for perishable goods such as meat, seafood, and dairy [62]. Smart packaging solves this through WSN and integrity indicators that monitor the “micro‐environment” inside each individual package [9]. An analytical review of these systems shows that they enable an operational redesign of the warehouse and transport systems [79]. For example, sensors that detect ammonia or CO_2_ release can trigger automated sorting systems to isolate compromised items before they contaminate a larger batch. Furthermore, the synergy between MAP and digital monitoring ensures that the protective gas composition is maintained, extending the shelf life of high‐value crops like spinach or berries [39]. This integrated approach moves food safety from a periodic lab‐based test to a continuous, automated process, ensuring that the final consumer receives products with preserved nutritional and organoleptic qualities [41].

#### 4.1.5. The Three‐Layer Architecture of Smart Packaging Systems

**Figure 8:**
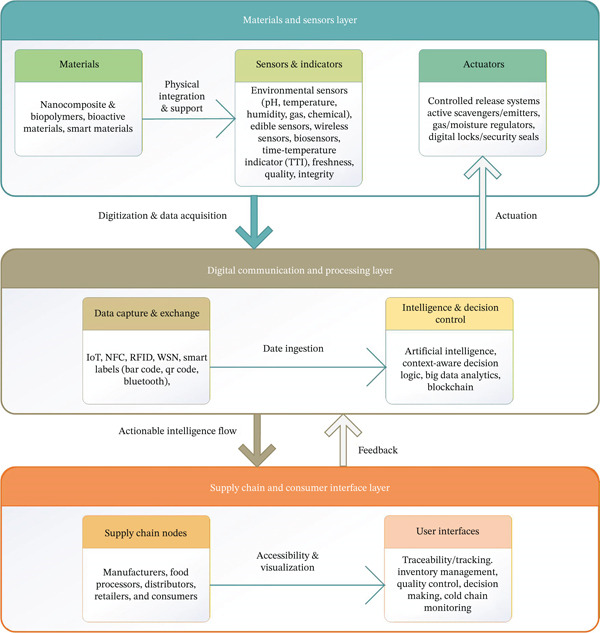
Three‐layer architecture in smart packaging for holistic, real‐time food quality management.

Figure 8 illustrates the functional integration of sensing, data processing, and logistical action within a conceptual three‐layer framework, which is fundamental to modern smart packaging solutions. This architecture is designed to facilitate holistic and real‐time management of food quality, incorporating a range of advanced technologies. Table 5 provides a detailed breakdown of the key technological components of this three‐layer structure, outlining their specific functions and contributions to smart packaging.

**Table 5:** Key technological components of three‐layer architecture and their functionality and contribution in smart packaging.

Layer	Primary focus	Key technological components	Functionality and contribution
Materials and sensors	Physical detection and sustainability	Biopolymers (chitosan, starch, PLA); nanocomposites (ZnO and nanocellulose); colorimetric indicators (anthocyanins and curcumin).	Detects physical/chemical degradation markers (pH, TVB‐N) nondestructively; provides sustainable alternatives to petroleum‐based plastics.
Digital communication and processing	Data intelligence and Security	IoT/RFID for continuous data acquisition; AI/ML for predictive modeling and dynamic shelf life; blockchain for immutable data integrity and traceability.	Converts raw sensor data into actionable intelligence; ensures secure, transparent, and real‐time data access for informed decisions.
Supply chain and consumer interface	Operational action and value realization	Adoption of technologies for FEFO logistics control; implementation of digital platforms for SCV; visual communication systems (e.g., color shift and NFC interface).	Reduces food waste by identifying spoilage earlier; increases product quality and safety assurance for both consumers and retailers.

This framework enables seamless interaction between materials, sensors, and digital communication systems, ensuring efficient monitoring and management of food products across the entire supply chain. The integration of these technologies empowers smart packaging to offer real‐time insights into food quality, enhance traceability, and support sustainability efforts in the packaging industry.

The table highlights the key technological components that drive the three‐layer architecture of smart packaging systems, showcasing their functionality and contributions in a streamlined and impactful way. First layer, materials and sensors, this layer revolves around material and sensor innovation, where the focus is on utilizing sustainable biopolymers and advanced nanocomposites, such as chitosan, starch, PLA, and ZnO. These materials enable the nondestructive detection of food degradation markers like pH and TVB‐N. In addition, innovative colorimetric indicators enhance real‐time quality monitoring while offering sustainable alternatives to traditional petroleum‐based plastics.

The second layer, digital communication and processing, this layer facilitates digital convergence and connectivity, turning raw sensor data into actionable insights. IoT, RFID, and blockchain technologies ensure continuous data acquisition, secure information sharing, and full traceability. AI and ML algorithms play a pivotal role in predicting food shelf life, enhancing decision‐making, and reducing waste. The integration of these digital tools ensures real‐time data flow and enhances the transparency and security of the supply chain.

The last layer, supply chain nodes and consumer interface. At the operational level, this layer focuses on operational supply chain and consumer interface integration. It empowers the supply chain through technologies like FEFO logistics control and digital platforms for supply chain visibility. On the consumer side, interfaces such as color‐shifting indicators and NFC tags provide immediate visual feedback on food quality, enabling smarter purchasing and consumption decisions.

These layers create a holistic approach to managing food quality, minimizing waste, and ensuring safety. The integration of sustainable materials, cutting‐edge digital tools, and consumer‐facing technology drives smarter, more transparent food packaging solutions that benefit both producers and consumers. The architecture exemplifies how innovation can streamline food supply chains and enhance real‐time food quality management.

### 4.2. Smart Food Packaging Regulations Across Countries and Sustainability Mandates

Innovations in smart and active packaging are driven by regulatory frameworks designed to ensure food safety, product quality, and sustainability. These regulations play a critical role in bridging the gap between laboratory innovations and their large‐scale commercial implementation [54]. As smart food packaging technologies rapidly evolve, there is an increasing need for a robust and harmonized regulatory framework that guarantees both safety and sustainability, especially as these technologies move from prototype stages to full‐scale commercial applications [80]. On a global scale, regulatory approaches vary significantly, typically categorized by philosophical orientation (positive list vs. exposure‐based risk) and the extent of technological integration (active and intelligent packaging) [81].

Table 6 provides a comparative analysis of smart food packaging regulations across different countries and regions, highlighting their regulatory philosophies, the status of smart packaging regulation, and the management of nanomaterial safety and migration. This table illustrates the diverse approaches to smart packaging regulation, offering insights into how different regions are addressing the challenges associated with these innovative technologies.

**Table 6:** Comparative analysis of smart food packaging regulations across countries.

Region/country	Regulatory philosophy and key focus	Status of smart packaging regulation	Nanomaterial and migration regulations
European Union (EU)	Follows a positive list approach, with a strong emphasis on consumer safety, quality, and organoleptic properties [81].	Regulated comprehensively under regulation (EC) No 1935/2004 and 450/2009. Emphasis on overall migration limits (OML) and specific migration limits (SML) [66].	Requires case‐by‐case approval for nanomaterials due to insufficient risk assessment protocols. Clear labeling required for nonedible components [80].
United States (US)	Employs an exposure‐based approach, focusing on estimated dietary intake (EDI). Relies on GRAS (generally recognized as safe) status [81, 82].	Regulated as food additives; PLA and chitosan from shrimp considered GRAS. Producers are encouraged to perform self‐reporting [60, 83].	Nanomaterials must be inert and not alter food quality. Nanomaterial definition based on final product properties [80].
Japan	Follows food sanitation laws and a positive list system. Prioritizes product quality [82, 84].	Regulated within the conventional food contact material legislation. Strict maximum residue limits for certain substances (e.g., 10 *μ*g/kg methylene blue in seafood) [54, 85].	Focused on industry standards; must comply with Container and Packaging Recycling Law [84].
China	Focuses on national standards such as GB 5009.228‐2016 to measure TVB‐N (total volatile basic nitrogen) as an indicator of spoilage [27].	Uses TVB‐N levels to assess sensor effectiveness; levels above 20 mg/100 g indicate spoiled fish. RFID/blockchain used for traceability [86].	Mandates standard FCM regulations (e.g., for plastic resins). Bans nondegradable plastic bags and excessive packaging [74].
South Korea	Strong focus on recycling and waste management. FCM regulated by the ministry of food and drug Safety (MFDS) [84].	Encourages real‐time quality monitoring (e.g., Kimchi).	Requires assessment and determination of packaging recycling levels (mandatory). Bans colored PET bottles and PVC use.
India	Regulated by the Food Safety And Standards Authority of India (FSSAI). Focus on extended producer responsibility (EPR) [84].	Most productive in global smart packaging research (119 documents, 1148 citations). Utilizes ICT technologies (e.g., e‐Choupal).	Allows recycled plastic (50% PCR) in FCM. Implements EPR and limits single‐use plastics.
Indonesia	No specific regulation for sp. Compliance with BPOM Regulation No. 20/2019. Total migration: 60 ppm or 10 mg/dm^2^ [87].	Smart components (sensors/indicators) treated as novel food contact substances requiring safety evaluation by BPOM.	Requires compliance with mandatory SNI for paper/carton (e.g., Pb limit 3 ppm).
Australia	Regulated by FSANZ (Food Standards Australia New Zealand). Focus on waste reduction (APCO) [84].	Regulated within conventional FCM legislation. Third largest market globally (projected $1.69 billion).	National Plastic Plan aims for 70% plastic recycling and 50% recycled content by 2025.
Other countries (Turkey, Iran, Brazil, Malaysia, Thailand, and Africa)	Varied, generally focused on EPR and banning single‐use plastics.	High research output in Iran (54 documents) and Turkey (27 documents). Brazil focuses on national solid waste policies and reverse logistics [84, 88].	Encourages bio‐based materials and biopolymers (e.g., Iran focuses on nanomaterial‐based films) [43].

#### 4.2.1. Regulatory Frameworks in Developed Markets (European Union [EU], United States, Japan, Australia, and South Korea)

The EU stands out for having the most comprehensive regulatory framework, governed by Regulation (EC) No 1935/2004 and Commission Regulation (EC) No 450/2009 [20]. The EU adopts a stringent, positive‐list‐based approach, with specific limits on overall migration limits (OMLs) and specific migration limits (SML) to prevent harmful substances from leaching into food [81]. The regulation of nanomaterials is particularly cautious, requiring case‐by‐case approval due to insufficient risk assessment protocols, with a default migration limit of $0.01 mg/kg for unapproved substances. European consumer concerns about “foreign substances” in packaging have also slowed adoption compared with other markets [20].

In contrast, the United States, regulated by the Food and Drug Administration (FDA), employs an exposure‐based risk approach [89]. Materials in contact with food are primarily regulated through the generally recognized as safe (GRAS) status, which is granted to substances deemed safe based on scientific evidence, such as chitosan and [81]. The FDA is more flexible with nanomaterials, lacking strict dimensional definitions, instead focusing on the inertness and nonalteration of food quality by the material. Additionally, the FDA issues no‐objection letters (NOLs) for recycled plastic processes, provided dietary exposure is considered negligible (1.5 *μ*g/person/day or $0.5 ppb) [74].

Japan and Australia are recognized for their leadership in the development of active and intelligent packaging technologies, often regulating these within the broader legislative framework for food contact materials (FCMs). Japan, for instance, sets maximum residue limits for certain substances, such as methylene blue (10 *μ*g/kg in seafood) [85]. South Korea emphasizes recycling, mandating compliance with the Resource Recycling Act and setting targets such as 30% recycled content in PET bottles by 2030 [84].

#### 4.2.2. Regulatory Frameworks in Asia and South America (China, India, Brazil, Iran, and Turkey)

China and India are major contributors to smart packaging research, leading in scientific publications [90]. China adheres to national standards like GB 5009.228‐2016 to measure decay indicators, such as TVB‐N, with values above 20 mg/100 g indicating spoiled fish [27]. India has a strong focus on extended producer responsibility (EPR) through the plastic waste management (amendment) rules, aiming to enhance recycling and sustainability [84].

Iran has also shown considerable research productivity, focusing on the development of active films and sensors using nanomaterials and natural dyes [56]. In Turkey, although there is significant consumer interest in innovative packaging, concerns about misleading information persist [20]. In South America, Brazil has adopted EPR principles and national solid waste policy to regulate packaging and promote reverse logistics, reflecting a trend toward more sustainable supply chain practices [74].

#### 4.2.3. Regulatory Frameworks in Southeast Asia and Africa

Southeast Asian countries, such as Malaysia and Thailand, are committed to reducing single‐use plastics (SUPs), as evidenced by the implementation of their Roadmap Toward Zero SUPs, encouraging the use of bio‐based materials [74]. In Africa, several countries, including Kenya, Uganda, Tanzania, and Tunisia, have taken steps to combat plastic pollution by banning single‐use plastic bags, though many still rely on traditional materials and face significant waste management challenges. In Africa, smart packaging research is largely in the laboratory stage, with Tunisia leading in the field [91].

#### 4.2.4. Regulatory Landscape in Indonesia

Indonesia presents a unique regulatory scenario where a comprehensive framework exists for general food and packaging safety, but no specific regulation addresses “smart food packaging” directly [87, 90]. Consequently, smart packaging must comply with existing food safety regulations, primarily under National Agency of Drug and Food Control (BPOM) Regulation No. 20/2019, which imposes strict requirements on FCMs and prohibits the use of materials that may pose health risks or release harmful substances. Components integral to smart packaging, such as sensors and smart materials, are categorized as novel food contact substances, requiring rigorous safety evaluations and approval by BPOM before use. The OML for such materials is typically set at 60 ppm or 10 mg/dm2. Furthermore, Indonesia mandates adherence to specific packaging quality control measures, including the National Standard of Indonesia (SNI) 8218:2015, which establishes limits on contaminants such as lead (Pb), with a maximum of 3 ppm in paper and carton products. This regulatory framework compels innovators to ensure the integrity of advanced materials, prevent biological contamination, and comply with transparency mandates for labeling and tracking technologies, enhancing traceability and consumer confidence [87].

This comparative analysis reveals the diverse regulatory landscapes that shape the development and adoption of smart food packaging worldwide. Although some regions, like the EU and United States, have established well‐defined frameworks for smart packaging regulation, others are still grappling with fragmentation and inconsistent standards. The varied market readiness and research outputs across countries also highlight the ongoing challenges in advancing these technologies globally. The common hurdles, including high costs, nanomaterial safety, and regulatory fragmentation, underscore the need for more harmonized international standards and increased investment in infrastructure to foster widespread adoption.

#### 4.2.5. Sustainability Mandates and Drivers

The sustainability mandates and drivers currently reshaping the food packaging industry are intricately linked to regulatory frameworks and legislative initiatives across major global regions, reflecting a concerted effort to address the dual imperatives of mitigating the global plastic waste crisis and substantially reducing food loss and waste (FLW), which accounts for approximately one‐third of global food production annually [74, 92]. This global push, particularly aligned with the UN Sustainable Development Goals (SDG 12), necessitates the technological transformation embodied by smart packaging solutions [6].

A critical sustainability driver involves the transition from petroleum‐based polymers to eco‐friendly alternatives, driven primarily by material innovation and governmental restrictions on SUPs [5]. The EU, for instance, spearheads this movement through directives like Regulation (EU) 2019/904, which restricts SUPs, simultaneously implementing ambitious recycling targets (e.g., 55% for plastic packaging by 2030) and mandating minimum recycled content in new plastic packaging (up to 30% for contact‐sensitive PET) [63, 84]. Similarly, several African countries, including Kenya and Tanzania, have adopted comprehensive national bans on SUP bags to curb widespread pollution, compelling manufacturers to seek sustainable material alternatives. In the United States, regulatory encouragement favors nontoxic, bio‐based polymers like PLA and chitosan, with the FDA granting them GRAS status for food contact applications [41, 60]. This shift promotes the use of natural biopolymers (starch, cellulose, and chitosan) derived from renewable resources or agricultural waste, which possess inherent properties like biodegradability and antimicrobial activity, further minimizing environmental impact [5, 29, 31].

Concurrently, the mandate to reduce FLW drives the integration of smart functionalities, necessitating regulatory validation of sensing components [6, 93]. FIs, often based on natural pigments like anthocyanins (from red cabbage or barberry) or curcumin, visually signal spoilage biomarkers (such as volatile basic nitrogen, or TVB‐N, compounds) by undergoing halochromic color shifts [19, 29]. This functionality is directly validated by national standards, such as China′s GB 5009.228‐2016, which defines fish spoilage criteria based on TVB‐N levels (typically 20 mg/100 g), thereby establishing clear performance benchmarks for smart sensors in high‐protein foods [33, 94]. However, countries like Indonesia maintain stringent control: Any sensor, indicator, or bioactive compound incorporated into the packaging is classified as a novel food contact substance and must undergo a mandatory safety assessment by BPOM, ensuring compliance with SML (OML, typically 10 mg/dm2) before large‐scale adoption, focusing on safety over speed of innovation [95]. The EU also strictly regulates the deployment of intelligent packaging, requiring adherence to Regulation (EC) No 450/2009 and requiring case‐by‐case approval for nanomaterials to prevent unforeseen migration risks [20, 54].

The digitization of the supply chain promotes sustainability through enhanced efficiency and compliance [9, 96]. Technologies such as IoT, RFID, and AI enable the transition from rigid inventory management (FIFO) to optimized FEFO strategies, directly curbing spoilage‐related food waste [11, 76]. This digital transparency is reinforced by mandatory EPR programs, prominently seen in India and Kenya, which hold producers accountable for the life‐cycle management of their packaging waste (including collection and recycling targets), promoting circular economy principles [84, 91]. Furthermore, research increasingly focuses on integrating recyclable electronics and edible sensors into packaging to mitigate the growing environmental impact of electronic waste associated with these digital components [73, 75, 97]. Thus, sustainability mandates across the globe compel innovative packaging design that not only ensures food safety and extends shelf life but also integrates seamlessly with stringent national and international regulatory expectations for consumer protection and environmental stewardship [66, 91].

### 4.3. Consumer Acceptance and Engagement in Smart Packaging

The rise of smart packaging technology is a direct response to the increasing consumer demand for safer, higher‐quality, fresh, and minimally processed food products. This shift underscores the critical role consumer perception plays in the commercial viability of these innovative systems [19]. Research has shown that a significant proportion of consumers are open to adopting SP technology; however, levels of acceptance often fluctuate depending on the specific functionality of the packaging and the perceived risks associated with it [98].

Consumer perceptions of smart packaging are largely influenced by the distinction between intelligent packaging and active packaging, with a notable preference for transparency and the immediacy of information. Consumers exhibit a favorable attitude toward intelligent packaging, which is defined by its ability to monitor the condition of food and provide real‐time information. This preference stems from the growing desire for dynamic updates on product quality, which addresses the inherent ambiguity of static “best before” or expiration dates [99, 100]. The real‐time insights offered by intelligent packaging allow consumers to make more informed decisions, enhancing their confidence in food quality. Intelligent packaging systems are highly valued for their nondestructive and instantaneous communication, often using simple visual signals such as colorimetric FIs [47]. For example, color changes in indicator films, caused by pH shifts during spoilage in protein‐rich foods, are seen as sensitive, straightforward, and effective tools for real‐time quality control by consumers [31]. These intuitive visual cues allow consumers to quickly assess food freshness without the need for complex testing or prolonged waiting periods.

In contrast, active packaging, which primarily functions by releasing active substances (e.g., antimicrobials or antioxidants) to extend the shelf life of food, tends to be viewed with greater skepticism. This apprehension arises from consumer concerns over the unintended migration of packaging components—such as dyes, active compounds, or nanomaterials—into the food product, potentially posing health risks [100]. This perception of risk significantly affects the willingness of consumers to accept active packaging technology, particularly when it comes to the safety and transparency of the materials involved [98]. The adoption of smart packaging technologies is also closely linked to digital literacy, consumer behaviors, and institutional trust. The success of smart packaging is increasingly dependent on how well these technologies integrate into the consumer′s digital ecosystem. The integration of NFC tags and RFID into packaging is particularly significant, as it facilitates user‐friendly interaction via smartphones [69]. By simply scanning an NFC tag with a smartphone, consumers can access detailed traceability data and product information, enhancing convenience and supporting informed purchasing decisions [100].

Consumers who are already engaged with existing packaging—such as those who regularly read ingredient labels or check expiry dates—are more likely to accept intelligent packaging technology [98]. This behavior suggests that clear, effective communication through packaging can facilitate acceptance, as consumers who prioritize product transparency are more inclined to adopt innovations that promise to enhance product safety and quality. A crucial factor in the acceptance of both intelligent packaging and active packaging technologies is the level of trust consumers have in the institutions overseeing food safety. Trust in governmental bodies, the agricultural sector, and consumer protection agencies plays a pivotal role in reducing the perceived risks associated with novel food technologies. When consumer trust is low, the likelihood of rejecting both intelligent packaging and active packaging increases, as uncertainty about the safety of these technologies remains high. Furthermore, manufacturers must ensure that nonedible components of packaging are clearly labeled to prevent consumer confusion and mitigate safety concerns [20, 98].

### 4.4. Challenges and Future Research

This bibliometric analysis highlights a critical transformation in the food packaging industry, where the integration of active packaging, intelligent packaging, and smart packaging is reshaping traditional packaging paradigms. However, it also outlines several significant challenges and areas that warrant further research and development.

#### 4.4.1. Scalability and Cost Effectiveness

One of the primary hurdles in the widespread adoption of smart packaging technologies is scalability. Although the technology has demonstrated significant advancements, transitioning from pilot projects to large‐scale manufacturing remains a challenge. This involves not only the integration of complex IoT systems but also the high costs associated with sensor production, the incorporation of sustainable materials, and the integration with existing packaging lines. As pointed out in the study by [14] and [2], whereas the current innovations in smart packaging show promise, the economic feasibility of scaling them across global food supply chains still requires substantial investment and technical refinement.

#### 4.4.2. Energy Demands and Sustainability Concerns

Another significant challenge is the energy consumption of IoT‐enabled devices, as highlighted by [101, 102]. Many smart packaging systems depend on embedded sensors and communication modules that require power, raising concerns about their long‐term sustainability. Future research should focus on developing energy‐efficient sensor systems or exploring alternative energy sources, such as energy harvesting technologies, which can generate power from environmental factors like temperature or motion changes. Additionally, integrating biodegradable and eco‐friendly materials remains a critical challenge in reducing the environmental impact of these technologies. This area is still in its early stages, particularly regarding large‐scale adoption, as discussed by [103, 104].

#### 4.4.3. Efforts Toward Regulatory Harmonization

The adoption of smart food packaging faces several universal challenges, including the high cost of sensors and the integration of Industry 4.0 systems, which impede the transition from prototypes to mass production at a commercially viable scale [105]. Additionally, concerns about the safety of nanomaterials, particularly the migration of organic and inorganic nanoparticles like ZnO and TiO_2_ into food, require more standardized analytical methods and stringent regulatory approval processes [80]. Furthermore, regulatory fragmentation, particularly between the EU′s positive list approach and the US′s exposure‐based model, creates trade barriers and escalates compliance costs, hindering the global adoption and harmonization of smart food packaging standards [74].

Despite these challenges, there is a global push for harmonization and sustainability. Organizations like Codex Alimentarius provide general guidelines on safety and labeling, and ongoing efforts are aimed at aligning FCM safety testing across the United States, EU, and Asia [74]. The integration of digital technologies (IoT, AI, and blockchain) is seen as key to enhancing traceability, reducing food waste, and verifying sustainability claims, which aligns with global regulatory goals.

This analysis highlights the diverse regulatory approaches and challenges surrounding the implementation of smart food packaging. Although significant progress has been made in certain regions, global harmonization remains a key challenge in advancing the widespread adoption of these innovative technologies.

#### 4.4.4. Consumer Acceptance and Engagement

Although smart packaging offers transparency and real‐time information on product freshness and quality, there is still a gap in consumer engagement and acceptance. Consumer engagement with technologies such as NFC and RFID is still limited, largely due to low awareness and initial implementation costs, as highlighted in previous studies [106, 107]. Future research should focus on increasing consumer awareness and clearly demonstrating the benefits of smart packaging, such as improved food safety and reduced waste, to encourage wider adoption. Additionally, simplifying the user experience through tools such as smartphone apps or easy‐to‐read labels will help increase engagement and make these technologies more accessible to the general public.

#### 4.4.5. Advanced Integration of AI, Big Data, and Blockchain

The integration of AI, big data, and blockchain technology will be crucial in advancing smart packaging, particularly in the food supply chain. Blockchain ensures transparent tracking and traceability by providing an immutable ledger of transactions, allowing stakeholders to monitor food products′ origin, handling, and storage in real time. This helps identify critical points where food loss occurs, improving quality control and reducing waste. Combining AI‐driven insights with blockchai′s secure data management can revolutionize supply chain operations, ensuring food safety and sustainability [45, 56]. Furthermore, integrating blockchain with IoT and RFID optimizes monitoring, whereas ongoing research into AI algorithms, ML, and DL, such as LSTM models, can enhance prediction accuracy and real‐time shelf‐life estimation, further mitigating FLW [12].

#### 4.4.6. Material Innovation and Sensor Development

The future of smart packaging is closely linked to advancements in materials science. The integration of biopolymers, biodegradable sensors, and nanocomposites has the potential to greatly enhance the functionality of smart packaging systems, as noted by [28, 34]. However, the development of these materials must focus on ensuring they are both cost‐effective and scalable for commercial use. Additionally, further research is needed into multifunctional materials that can integrate multiple types of sensors (e.g., pH, gas, and temperature) into a single packaging solution, enabling comprehensive monitoring without significantly increasing production costs.

## 5. Conclusion

Smart Packaging 4.0 integrates sensors and digital technologies to enhance food safety, shelf‐life monitoring, and supply chain traceability, ultimately reducing food waste and fostering sustainability. This innovation relies on advanced sensors, real‐time monitoring, and AI‐driven predictive models to improve food quality management, providing accurate, data‐driven insights for better storage and transport conditions. Moreover, the focus on eco‐friendly materials, including biodegradable polymers and bio‐based sensors, contributes to environmental sustainability by reducing the impact of traditional packaging methods. These technologies are aimed at making food packaging more interactive and responsive, actively preserving food quality through real‐time monitoring and enhancing consumer transparency.

However, several limitations challenge the widespread adoption of Smart Packaging 4.0. Scalability remains a significant hurdle, as transitioning from experimental prototypes to large‐scale production involves high costs and complex integration into existing packaging processes. The energy demands of devices, such as sensors and communication modules, raise concerns about sustainability, particularly for long‐term use. Additionally, regulatory challenges regarding data security and the lack of standardized global regulations complicate the implementation of these technologies. Consumer engagement with smart packaging is also limited, primarily due to low awareness and the high initial costs. Furthermore, the development of biodegradable and multifunctional materials is hindered by scalability and cost issues, making it difficult to incorporate these innovations into mainstream applications.

## Author Contributions

M.J.D., S.J.M., and H.M.E. contributed to the conceptualization, methodology, data curation, software, visualization, and drafting and editing of the original manuscript. H.M.E., S.J.M., J.F., and S.E.Y.T. were involved in providing formal analysis and validation, and contributed to the manuscript′s preparation and critical review. L.L. and N.N. provided supervision, performed formal analysis, interpreted data, and critically reviewed the manuscript for intellectual content. S.S., W.P., and B.S.S. offered validation, critically reviewed the manuscript, and contributed to editing.

## Funding

No funding was received for this manuscript.

## Disclosure

All authors unanimously approved the final version and agreed to be accountable for all aspects of the work, ensuring that questions regarding its accuracy or integrity are properly addressed.

## Conflicts of Interest

The authors declare no conflicts of interest.

## Data Availability

The data that support the findings of this study are openly available in Mendeley Data at https://data.mendeley.com/datasets/, Reference Number 10.17632/jc6hx5xxpz.1.
